# Antihyperglycemic Activity of *Houttuynia cordata* Thunb. in Streptozotocin-Induced Diabetic Rats

**DOI:** 10.1155/2014/809438

**Published:** 2014-02-24

**Authors:** Manish Kumar, Satyendra K. Prasad, Sairam Krishnamurthy, Siva Hemalatha

**Affiliations:** Pharmacognosy Research Laboratory, Department of Pharmaceutics, Indian Institute of Technology, Banaras Hindu University, Varanasi 221005, India

## Abstract

Present study is an attempt to investigate plausible mechanism involved behind antidiabetic activity of standardized *Houttuynia cordata* Thunb. extract in streptozotocin-induced diabetic rats. The plant is used as a medicinal salad for lowering blood sugar level in North-Eastern parts of India. Oral administration of extract at 200 and 400 mg/kg dose level daily for 21 days showed a significant (*P* < 0.05) decrease in fasting plasma glucose and also elevated insulin level in streptozotocin-induced diabetic rats. It also significantly reversed all the alterations in biochemical parameters, that is, total lipid profile, blood urea, creatinine, protein, and antioxidant enzymes in liver, pancreas, and adipose tissue of diabetic rats. Furthermore, we have demonstrated that the extract significantly reversed the expression patterns of various glucose homeostatic enzyme genes like GLUT-2, GLUT-4, and caspase-3 levels but did not show any significant effect on PPAR-**γ** protein expressions. Additionally, the extract positively regulated mitochondrial membrane potential and succinate dehydrogenase (SDH) activity in diabetic rats. The findings justified the antidiabetic effect of *H. cordata* which is attributed to an upregulation of GLUT-4 and potential antioxidant activity, which may play beneficial role in resolving complication associated with diabetes.

## 1. Introduction

Diabetes mellitus (DM) is a disease that results in chronic inflammation and apoptosis in pancreatic islets in patients with either type 1 or 2 DM and is characterized by abnormal insulin secretion [1]. Insulin-resistant glucose use in peripheral tissues such as muscle and adipose tissues is a universal feature of both insulin-dependent DM and non-insulin-dependent DM. In this process, glucose transporters (GLUTs) play crucial role [2]. Glucose transporter 4 is mainly expressed in skeletal muscle, heart, and adipose tissues which plays critical role in insulin stimulated glucose transport in these tissues, with glucose uptake occurring when insulin stimulates the translocation of GLUT-4 from the intracellular pool to the plasma membrane [3]. Glucose transporter 2, being the primary GLUT isoform in the liver, plays a pivotal role in glucose homeostasis by mediating bidirectional transport of glucose [4]. It is reported that oxidative stress plays a major role in the development of diabetes associated disorders, possibly due to overproduction of reactive oxygen species (ROS) [5]. Glucose and lipid metabolism are largely dependent on the mitochondrial functional state and physiology which, on excessive ROS formation, leads to mitochondrial oxidative damage and reduced mitochondria biogenesis that contributes to insulin resistance and associated diabetic complications [6, 7].

Medicinal plants continue to be an important source in search of a suitable active principle(s), wherein they are currently being investigated for their potential pharmacological properties in the regulation of conditions such as elevated blood glucose level in diabetes [8]. *Houttuynia cordata* Thunb. (HC) is a single species of its genus and is native to Japan, South-East Asia, and Himalayas. Ethnomedically, whole plant of *H. cordata* is being used for the treatment of diabetes. In the Ri-Bhoi district of Meghalaya, India, whole plant of *H. cordata *is eaten raw as a medicinal salad for lowering the blood sugar level and is commonly known by the name Jamyrdoh [9, 10]. The plant is also used as an ingredient in insulin secretion promoter compositions [11]. In southern China, green leaves and young roots are used as vegetable while dry leaves are used to prepare drink by boiling decoction [12, 13]. Reported pharmacological activities of plant including hypoglycaemic [14], antileukemic [15], anticancer [16], adjuvanticity [14], antioxidant [17] and inhibitory effects on anaphylactic reaction and mast cell activation [16].

A recent study has shown that the volatile oil from *H. cordata* restored the alterations in blood glucose, insulin, adiponectin, and connective tissue growth factor levels in diabetic rats, induced by the combination of a high-carbohydrate and high-fat diet, and STZ injection which may be attributed to the reduced insulin resistance, adiponectin, and connective tissue growth factor levels [18]. Jang et al. [19] reported the potential advanced glycation end products formation and rat lens aldose reductase inhibitory activity of two flavonol rhamnosides 4 and 5 isolated from the whole plant of* H. cordata*. On the basis of the above reports, the present study was undertaken for the first time to assess the mechanism involved in protective role of *H. cordata* against STZ-induced glucose toxicity using rat liver, pancreas, and adipose tissue as the working model. In addition, a mechanistic approach of *H. cordata* against STZ-induced inflammation, apoptosis, and mitochondrial dysfunction was proposed for evaluation. Moreover, GLUT-2 in liver and pancreas and GLUT-4 in adipose tissue were expressed to explain the probable mechanism of *H. cordata* against STZ-induced impaired glucose utilization.

## 2. Material and Methods

### 2.1. Chemicals Used

Streptozotocin, tetra methyl rhodamine methyl ester (TMRM), glutamate, and *o*-phthalaldehyde (OPA) were procured from Sigma-Aldrich (St. Louis, MO, USA). Glibenclamide (standard drug) was obtained as a gift sample from Accent pharma (QC. Ref. No. GLB/B129/10/11) Pvt. Ltd. Thiobarbituric acid (TBA), ethylene glycol tetra-acetic acid (EGTA), and 2-[4-(2-hydroxyethyl)1-piperazinyl]ethanesulfonic acid (HEPES buffer, acid free) were purchased from Hi-media (Mumbai) and sodium succinate, sodium azide, phenazine methanesulphonate (PMS), and nitro blue tetrazolium were purchased from Merck (Darmstadt, Germany). All other chemicals and reagents were procured from local suppliers and were of analytical grade. Plasma insulin was assayed by using commercial enzyme-linked immunosorbent assay kit (ELISA, Boerhringer Mannheim, Germany).

Total cholesterol (TC), high density lipoprotein (HDL) cholesterol, triglyceride (TG), blood urea nitrogen (BUN), creatinine (CRTN), and total protein (TPR) were estimated using kits from Span Diagnostics Ltd., India.

### 2.2. Animals

Albino rats of Charles foster strain with body weights of (160–200 g) were obtained from the Central Animal House (Registration number: 542/02/ab/CPCSEA), Institute of Medical Science (IMS), Banaras Hindu University (BHU), Varanasi, India. Before and during the experiment, rats were fed with normal laboratory pellet diet (Hindustan lever Ltd., India) and water *ad libitum*. After randomization into various groups, the rats were allowed to acclimatize for a period of 2-3 days in the new environment before initiation of experiment. The experimental protocol has been approved by the institutional animal ethics committee (Reference number Dean/10-11/58 dated 07.03.2011).

### 2.3. Plant Material and Preparation of Extract

*Houttuynia cordata* Thunb. herb was collected from Jaintia Hills of Meghalaya, India. The plant was identified by Dr. B. K. Sinha, Botanical Survey of India. A voucher specimen number (COG/HC/011) was deposited in the Department of Pharmaceutics, Indian Institute of Technology, Banaras Hindu University, Varanasi (U.P), India. Whole plants of *H. cordata* were washed with water and after being shade dried, the plant material was ground in a mill, passed through sieve number 40 to obtain a coarse plant powder. Dried powdered material (1 kg) of whole plant of *H. cordata *was extracted with 3 liter ethanol by soxhlation for 6 h. The resulting extract was concentrated under reduced pressure to obtain a dark crude residue (yield: 13.2% w/w).

### 2.4. Phytochemical Analysis

The extract was subjected to various phytochemicals tests to determine the active constituents present in the crude ethanolic of *H. cordata* [20]. Total phenolic and tannin content in *H. cordata* was estimated according to the method of Makkar, [21] using Folin ciocalteu reagent, whereas the method proposed by Kumaran and Joel Karunakaran [22] was followed to estimate total flavonoid and flavonol contents in *H. cordata*.

Further, *H. cordata* was standardized with quercetin using high performance thin layer chromatography (HPTLC). A stock solution of both *H. cordata* and standard quercetin in methanol was prepared in concentration of 5 mg/mL and 0.2 mg/mL respectively. Mobile phase for developing the chromatogram was composed of chloroform: methanol and formic acid mixture in the ratio 7.5 : 1.5 : 1 (v/v/v). The study was carried out using Camag-HPTLC instrumentation equipped with Linomat V sample applicator, Camag TLC scanner 3, Camag TLC visualizer, and WINCATS 4 software for data interpretation. The *R*_*f*_ values were recorded and the developed plate was screened and photo-documented at ultraviolet range with wavelength (*λ*_max⁡_) of 254 nm.

### 2.5. Oral Toxicity Studies

Acute oral toxicity study of ethanolic extract from *H. cordata* was done according to “Organization for Environmental Control Development” guidelines (OECD: Guidelines 425; Up and Down Procedure). The study was performed on 24 h fasted rats by single dose administration each of 2000 and 5000 mg/kg, (p.o.). The toxicity sign and symptoms or any abnormalities associated with the ethanolic extract of *H. cordata *were observed at 0, 30, 60, 180, and 240 min and then once a day for the next 14 days. The number of rats that survived was recorded at the end of the study period.

### 2.6. Induction of Experimental Diabetes

The animals were fasted overnight and diabetes was induced by a single intraperitoneal injection of a freshly prepared solution of streptozotocin (65 mg/kg, b.w.) in 0.1 M citrate buffer (pH 4.5) [23]. The animals were allowed free access to 5% glucose solution to overcome the drug induced hypoglycemia. Diabetes was confirmed after 48 h and then on the 7th day of streptozotocin injection, the blood samples were collected through retroorbital venous plexus under light anesthesia and plasma glucose levels were estimated by enzymatic GOD-PAP (glucose oxidase peroxidase) diagnostic kit method. The rats having fasting plasma glucose (FPG) levels more than 200 mg/dL were selected and used for the present study [24].

### 2.7. Experimental Design

The diabetic animals were divided into six groups (*n* = 6). Group-I, normal control (untreated) rats; group-II, diabetic control rats; group-III, diabetic rats given glibenclamide 10 mg/kg orally for 21 days; group-IV, group-V, and group-VI, diabetic rats that received *H. cordata* extract at 100, 200, and 400 mg/kg, p.o. body weight, respectively, once daily for 21 days. At the 0th, 7th, 14th, and 21st days blood from each rat was collected through retroorbital venous plexus under light anesthesia. Plasma was separated and the FPG level was estimated. Plasma lipid profile (TC, TG, LDL, HDL, and VLDL), insulin, and other biochemical parameters, that is, creatinine (CRT), blood urea nitrogen (BUN), and total protein (TPR), were also estimated on the 21st day of the experiment.

### 2.8. Evaluation of Mitochondrial Function and Oxidative Stress

#### 2.8.1. Mitochondria Isolation Procedure

Mitochondria were isolated by standard differential centrifugation [25]. The liver, pancreas, and adipose tissue were homogenized in (1 : 10, w/v) ice cold isolation buffer (250 mM sucrose, 1 mM EGTA, and 10 mM HEPES-KOH, pH 7.2). Homogenates were centrifuged at 600 ×g/5 min and the resulting supernatant was centrifuged at 10,000 ×g/15 min and supernatant discarded. Pellets were next suspended in medium (1 mL) consisting of 250 mM sucrose, 0.3 mM EGTA, and 10 mM HEPES-KOH, pH 7.2 and again centrifuged at 14,000 ×g/10 min. All centrifugation procedures were performed at 4°C. The final mitochondrial pellet was resuspended in medium (1 mL) containing 250 mM sucrose and 10 mM HEPES-KOH, pH 7.2, and used within 3 h. Mitochondrial protein content was estimated using the method of Lowry et al. [26].

#### 2.8.2. Estimation of Mitochondrial Antioxidant Enzymes

Mitochondrial malondialdehyde (MDA) content was measured based on the TBA reaction test [27]. The activity of superoxide dismutase (SOD) was assayed by the method of Kakkar et al. based on the formation of NADH-phenazine methosulphate-nitro blue tetrazolium formazan measured at 560 nm against butanol as blank [28]. Decomposition of hydrogen peroxide in presence of catalase (CAT) was followed at 240 nm [29]. The results were expressed as units (U) of CAT activity/min/mg of protein.

#### 2.8.3. Estimation of Mitochondrial Succinate Dehydrogenase Activity (SDH)

The mitochondrial succinate: acceptor oxidoreductase (EC 1.3.99.1) was determined by standard protocol based on the progressive reduction of NBT to an insoluble colored compound [a diformazan (dfz)] used as a reaction indicator [30]. The reaction of NBT was mediated by H^+^ released in the conversion of succinate to fumarate. The concentration of NBT-dfz produced was measured at 570 nm. The mean SDH activity of each region was expressed as micromole formazan produced per min per microgram of protein.

#### 2.8.4. Estimation of Mitochondrial Membrane Potential (MMP)

The Rhodamine dye taken up by healthy mitochondria was measured by fluorometric methods [31]. The mitochondrial suspension was mixed with TMRM solution. The mixture was then incubated for 5 min at 25°C temperature and any unbound TMRM was removed by frequent washings (four times). Then the buffer was added to make up the final volume and florescence emission was read at an excitation *λ*  535 ± 10 nm and emission *λ* of 580 ± 10 nm using slit number 10. The peak fluorescence intensity recorded was around *λ*  570 ± 5 nm. The results are expressed as fluorescence intensity value per milligram of protein.

### 2.9. Evaluation of Caspase-3, PPAR-*γ*, GLUT-2, and 4 by Western Blotting

Caspase-3, PPAR-*γ*, GLUT-4, and 2 antibodies were purchased from Santa Cruz Biotechnology Inc (Santa Cruz, CA, USA). The liver and pancreatic tissues were homogenized in lysis buffer and centrifuged. Lysates (80 *μ*g proteins) were electrophoresed on 10% sodium dodecyl sulfate (SDS)-PAGE gels and then transferred to polyvinyl difluoride (PVDF) membranes (Bio-Rad, USA). The membranes were incubated with rabbit polyclonal anti-caspase-3 antibody (1 : 1000), rabbit polyclonal anti-GLUT-4, and anti-GLUT-2 antibody (1 : 2000 and 1 : 1500) or mouse monoclonal anti-PPAR-*γ* antibody (1 : 1000) overnight at 4°C, and then with horseradish peroxidise-conjugated goat anti-rabbit IgG (1 : 3000) or horseradish peroxidise-conjugated goat anti-mouse IgG (1 : 3000) for 60 min at room temperature. Western blotting luminescent reagent was used to visualize peroxidase activity. Normalization was carried out by stripping films and reprobing with a mouse monoclonal antibody to the *β*-isoform of actin (1 : 10000, Sigma). Films were scanned and subsequently analyzed by measuring optical densities of immunostained bands using an image processing and analysis system (Image J 1.37 software, NIH, USA).

### 2.10. Pancreatic Histology

For histopathological studies the pancreas was blotted, dried, and fixed in 10% formalin for 48 h. Thereafter, the tissues were dehydrated in acetone for 1 h and embedded in paraffin wax. Section of pancreatic tissues was then taken through microtome and stained with Haematoxylin-Eosin for photomicroscopic observation [32], which was carried out on Nikon Trinocular Microscope, Model E-200, Japan.

### 2.11. Statistical Analysis

The data were analyzed with GraphPad Prism version 5 (San Diego, CA). Statistical analysis was done by two-way ANOVA, followed by Bonferroni posttest for FPG, whereas other biochemical parameters were analysed by one-way ANOVA, followed by Tukey's multiple comparison test. Data are expressed as mean ± SEM. A level of *P* < 0.05 was accepted as statistically significant.

## 3. Results

### 3.1. Phytochemical Analysis

**Figure 1:**
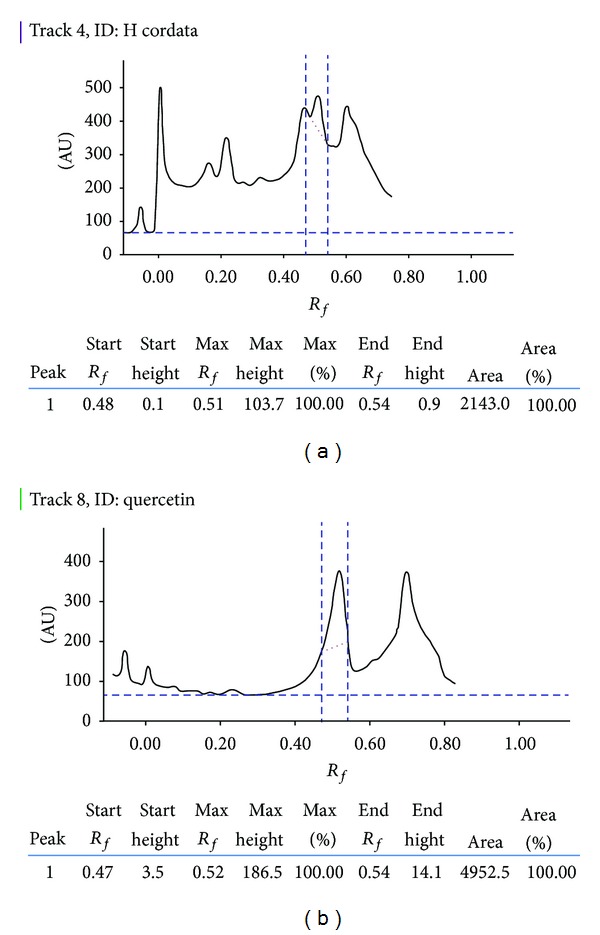
HPTLC densitogram of quercetin in ethanolic extract of *H. cordata* (HC). (a) Peak of quercetin present in HC and (b) standard peak of quercetin.

Preliminary phytochemical analysis of the extract revealed the presence of phenols, flavonoids, tannins, alkaloids, steroids, and carbohydrates as a major component. Total phenolic content of *H. cordata* was reported to be 45.74 mg/g gallic acid equivalent while total tannin content was estimated to be 33.29 mg/g tannic acid equivalent. Total flavonoid and flavonol contents were found to be 104.55 and 17.16 mg/g rutin equivalents. HPTLC studies revealed well-resolved peaks of *H. cordata* containing quercetin. The spots of the entire chromatogram were visualized under UV 254 nm and the percentage of quercetin (*R*_*f*_ 0.51) in *H. cordata* extract was found to be 4.39% (w/w) (Figure 1).

### 3.2. Effects of *H. cordata* on FPG Levels in STZ-Induced Diabetic Rats

Table 1 demonstrates a time-dependent effect on the level of FPG in STZ-induced diabetic rats showing significant interaction between groups ([*F*(5,15) = 10.16, *P* < 0.05] and days [*F*(3, 15) = 1.92, *P* < 0.05]. Statistical analysis by two-way ANOVA revealed that there was no significant difference among the groups at the 0th and 7th days except glibenclamide (10 mg/kg, p.o.) treated rats. However, statistical analysis at the 14th and 21st days revealed a significant reduction in the plasma sugar level of glibenclamide and *H. cordata* (200 and 400 mg/kg, p.o.) treated groups compared to diabetic control groups.

### 3.3. Effect on Plasma Lipid Profile and Other Biochemical Parameters

The effect of *H. cordata* on TC, TG, LDL, HDL, and VLDL is represented in Table 2. The results demonstrated a significant decrease in TC [*F*(5, 30) = 29.24, *P* < 0.05], TG [*F*(5, 30) = 25.75, *P* < 0.05], LDL [*F*(5,30) = 37.98, *P* < 0.05] and VLDL [*F*(5,30) = 25.75, *P* < 0.05] levels in glibenclamide and *H. cordata* (200 and 400 mg/kg, p.o.) treated groups. Moreover, glibenclamide and *H. cordata* also showed significant increase in HDL level [*F*(5,30) = 33.29, *P* < 0.05]. The results also depicted a significant reduction in total creatinine [*F*(5,30) = 7.1, *P* < 0.05] and blood urea nitrogen [*F*(5, 30) = 21.46, *P* < 0.05], content at 200 and 400 mg/kg, p.o. of *H. cordata*; however, a significant increase in total protein [*F*(5,30) = 21.58, *P* < 0.05] was observed only at 400 mg/kg, p.o. of *H. cordata* (Table 3).

### 3.4. Effect on Body Weight

Table 4 represents the effect of *H. cordata* on body weight of treated rats. Although the mean body weight of treated groups (100 and 200 mg/kg; p.o.) was higher than diabetic control group, it was not statistically significant. However, the rats treated with glibenclamide (10 mg/kg; p.o.) and *H. cordata* (400 mg/kg, p.o.) showed significant increase in body weight compared to diabetic control.

### 3.5. Effect on Insulin Levels

**Figure 2:**
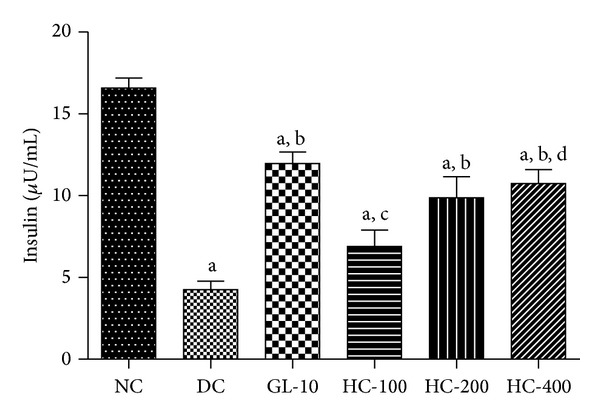
Effect of HC on plasma Insulin levels in control and experimental rats. Values are given as mean ± SD, *n* = 6, ^a^ *P* < 0.05 compared to normal control; ^b^ *P* < 0.05 compared to diabetic control; ^c^ *P* < 0.05 compared to glibenclamide; ^d^ *P* < 0.05 compared to HC 100 (one-way ANOVA followed by Tukey's multiple comparison test).

The effect of *H. cordata* (100, 200 and 400 mg/kg, p.o.) on plasma insulin is depicted in Figure 2. Post hoc test revealed a significant reduction in plasma insulin level in diabetic rats compared to normal control. However, glibenclamide and *H. cordata* at 200 and 400 mg/kg, p.o. showed significant [*F*(5, 30) = 23.94, *P* < 0.05] increase in plasma insulin levels compared to diabetic control.

### 3.6. Effect of *H. cordata* on Mitochondrial Antioxidant Level

One-way ANOVA showed that, in liver [*F*(5,30) = 21.58, *P* < 0.05], pancreas [*F*(5,30) = 53.6,  *P* < 0.05], and adipose tissue [*F*(5,30) = 47.39, *P* < 0.05], there were significant differences in MDA levels among groups (Table 5). Post hoc analysis revealed that hyperglycaemia significantly increased MDA levels compared to normal control. However, treatment with *H. cordata* (200 and 400 mg/kg, p.o.) reversed diabetes-induced MDA levels significantly in all the regions. The changes in mitochondrial SOD activity as a measure of mitochondrial antioxidant function are represented in Table 5. Analysis by one-way ANOVA showed that there was significant difference in the SOD activity in liver [*F*(5, 30) = 7.31, *P* < 0.05], pancreas [*F*(5,30) = 9.19, *P* < 0.05], and adipose tissue [*F*(5,30) = 8.04, *P* < 0.05] among groups. However, administration of* H. cordata* (200 and 400 mg/kg, p.o.) significantly increased the SOD activity in all the three tissues. Statistical analysis by one-way ANOVA showed that there was significant difference in the CAT activity in liver [*F*(5,30) = 11.13, *P* < 0.05], pancreas [*F*(5,30) = 5.84, *P* < 0.05], and adipose tissue [*F*(5,30) = 18.83, *P* < 0.05] among groups. Nevertheless, treatment with *H. cordata* (200 and 400 mg/kg, p.o.) significantly increased the CAT activity in all three regions compared to diabetic control groups.

### 3.7. Effect of *H. cordata* Extract on Mitochondrial Function

The mitochondrial function in terms of mitochondrial SDH activity was determined in STZ-induced diabetic animals (Table 5). One-way ANOVA showed that there were significant differences in mitochondrial function among groups in pancreas [*F*(5,30) = 34.81, *P* < 0.05], liver [*F*(5,30) = 46.93, *P* < 0.05], and adipose tissue [*F*(5,30) = 23.38, *P* < 0.05]. Post hoc analysis revealed that the mitochondrial function was decreased in terms of decrease in the mitochondrial SDH activity in all the tissues of STZ-induced rats compared to control animals. *H. cordata* (200 and 400 mg/kg, p.o.) significantly reversed STZ-induced decrease in mitochondrial function in pancreatic tissues.

### 3.8. Effect of *H. cordata* Extract on Mitochondrial Membrane Potential (ΔΨ_*m*_)

**Figure 3:**
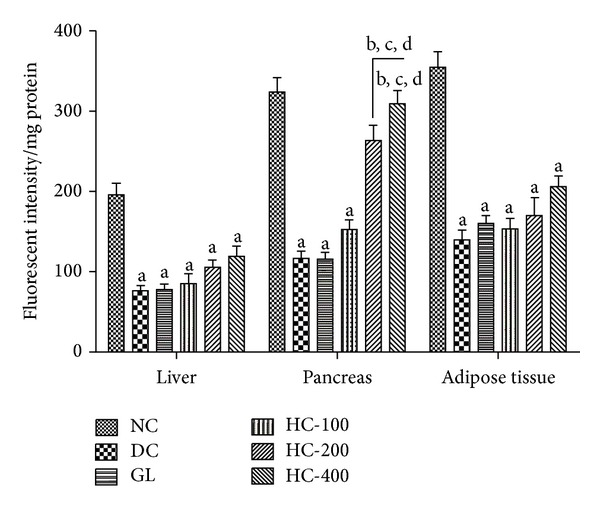
Effect of HC on MMP levels in liver, pancreas and adipose tissue in STZ-induced diabetic rats. Bars represent data as mean ± SEM, *n* = 6, ^a^ *P* < 0.05 compared to normal control; ^b^ *P* < 0.05 compared to diabetic control; ^c^ *P* < 0.05 compared to glibenclamide; ^d^ *P* < 0.05 compared to HC 100 (one-way ANOVA followed by Tukey's multiple comparison test).

The changes in ΔΨ_*m*_ as a marker of mitochondrial integrity during the hyperglycaemic condition are represented in Figure 3. One-way ANOVA showed that there were significant differences in mitochondrial integrity among groups in pancreas [*F*(5,30) = 41.95, *P* < 0.05], adipose tissue [*F*(5,30) = 25.26, *P* < 0.05], and liver [*F*(5,30) = 18.41, *P* < 0.05]. Post hoc analysis showed that STZ caused loss in the mitochondrial integrity in all the tissues compared to control rats. *H. cordata* (200 and 400 mg/kg, p.o.) significantly mitigated mitochondrial integrity in pancreas compared to diabetic control. Glibenclamide treatment also showed no significant effect on diabetes-induced decline in ΔΨ_*m*_ in all the regions under the investigation.

### 3.9. Effect on Apoptosis

**Figure 4:**
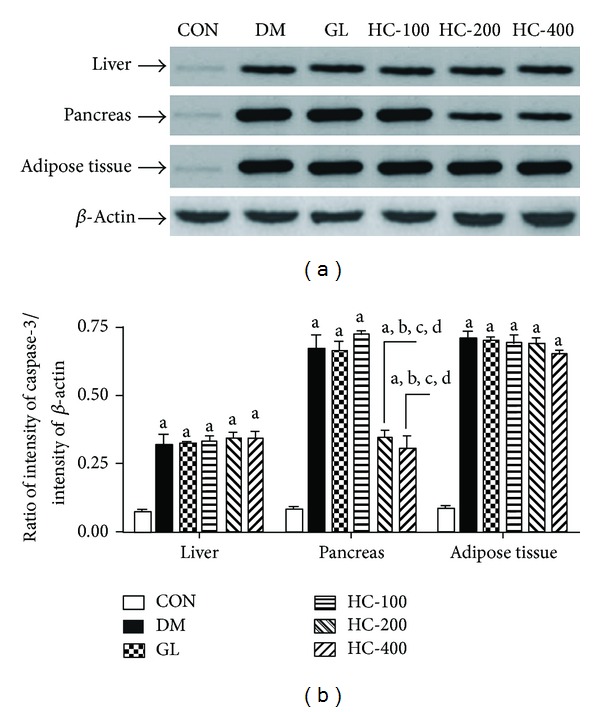
Effect of HC (100, 200 and 400 mg/kg) on STZ-induced changes in the levels of expression of caspase-3 in liver, pancreas, and adipose tissues. The blots (a) are representative of caspase-3 in liver, pancreas, and adipose tissues. The results in the histogram (b) are expressed as ratio of relative intensity of levels of protein expression of caspase-3 to *β*-Actin. All values are mean ± SEM of three separate sets of independent experiments. ^a^ *P* < 0.05 compared to normal control; ^b^ *P* < 0.05 compared to diabetic control; ^c^ *P* < 0.05 compared to glibenclamide; ^d^ *P* < 0.05 compared to HC 100; ^e^ *P* < 0.05 compared to HC 200 (One-way ANOVA followed by Tukey's multiple comparison test).

Figure 4 illustrates the effect of *H. cordata* on STZ-induced changes in the level of caspase-3 as a marker of apoptosis in liver, pancreas, and adipose tissues. One-way ANOVA revealed that there were significant differences in the level of expression of caspase-3 among groups in pancreas [*F*(5,12) = 50.19, *P* < 0.005], liver [*F*(5,12) = 27.37, *P* < 0.005] and adipose tissues [*F*(5,12) = 11.32, *P* < 0.005]. Post hoc analysis showed that *H. cordata* (200 and 400 mg/kg, p.o.) significantly reversed the STZ-induced apoptosis in pancreas only.

### 3.10. PPAR-*γ* Expressions in Liver, Pancreas, and Adipose Tissue

**Figure 5:**
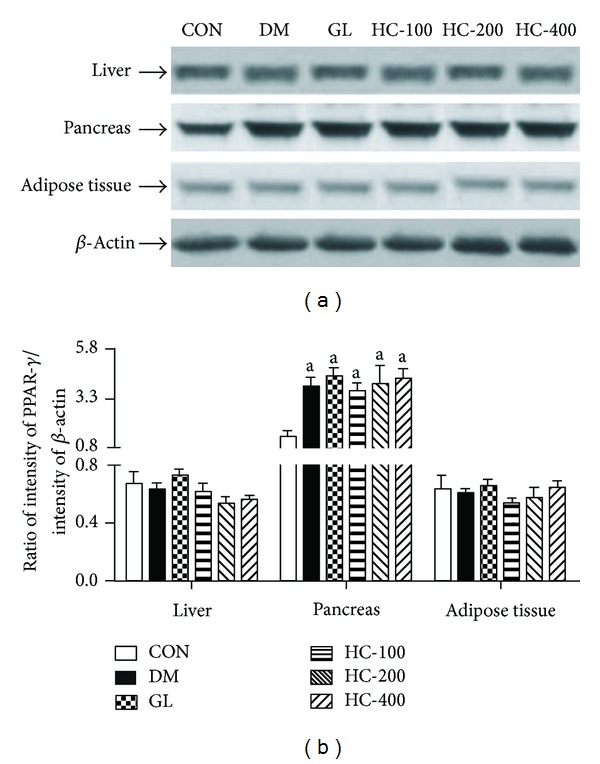
Effect of HC (100, 200 and 400 mg/kg) on STZ-induced changes in the levels of expression of PPAR-**γ** in liver, pancreas, and adipose tissues. The blots (a) are representative of PPAR-**γ** in liver, pancreas, and adipose tissues. The results in the histogram (b) are expressed as ratio of relative intensity of levels of protein expression PPAR-**γ** to *β*-Actin. All values are mean ± SEM of three separate sets of independent experiments. ^a^ *P* < 0.05 compared to normal control (one-way ANOVA followed by Tukey's multiple comparison test).

Figure 5 shows PPAR-*γ* expressions as a marker of inflammation in all three regions of normal control, diabetic control, and diabetic rats subjected to glibenclamide and *H. cordata* treatment after 21 days. The level of PPAR-*γ* expression was significantly increased among the groups in the pancreas [*F*(5,12) = 6.51, *P* < 0.005]; however, there was no significant change observed in liver [*F*(5,12) = 1.93, *P* < 0.005] and adipose tissue [*F*(5,12) = 0.79, *P* < 0.005] compared to normal control rats. Further, post hoc analyses revealed that glibenclamide and *H. cordata* had no significant effect on PPAR-*γ* expression.

### 3.11. Effect of *H. cordata* on GLUT-2 in Liver and Pancreas and GLUT-4 in Adipose Tissue

**Figure 6:**
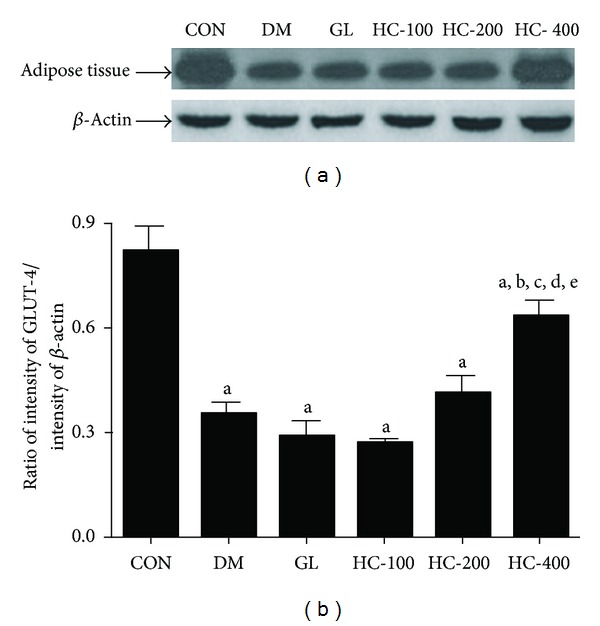
Effect of HC (100, 200 and 400 mg/kg) on STZ-induced changes in the levels of expression of GLUT-4 in adipose tissues. The blots (a) are representative of GLUT-4 in adipose tissues. The results in the histogram (b) are expressed as ratio of relative intensity of levels of protein expression of GLUT-4 to *β*-Actin. All values are mean ± SEM of three separate sets of experiments. ^a^ *P* < 0.05 compared to normal control; ^b^ *P* < 0.05 compared to diabetic control; ^c^ *P* < 0.05 compared to glibenclamide; ^d^ *P* < 0.05 compared to HC 100; ^e^ *P* < 0.05 compared to HC 200 (one-way ANOVA followed by Tukey's multiple comparison test).

**Figure 7:**
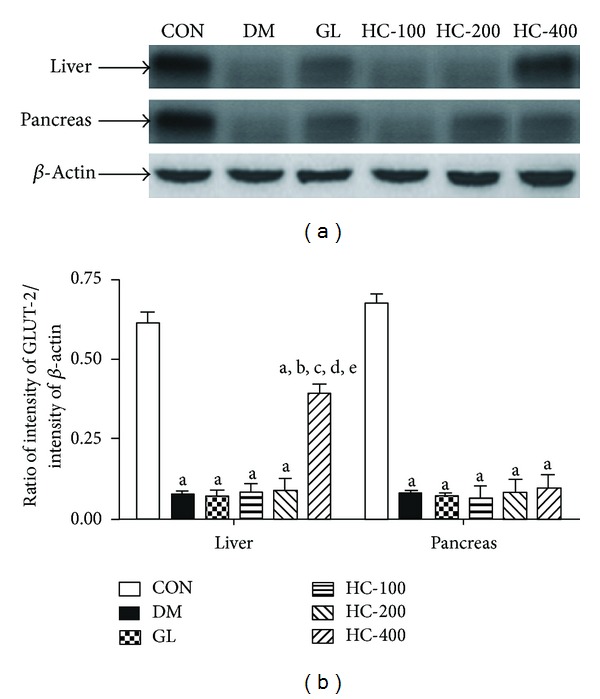
Effect of HC (100, 200 and 400 mg/kg) on STZ-induced changes in the levels of expression of GLUT-2 in liver and pancreas. The blots (a) are representative of GLUT-2 in liver and pancreas. The results in the histogram (b) are expressed as ratio of relative intensity of levels of protein expression of GLUT-2 to *β*-Actin. All values are mean ± SEM of three separate sets of independent experiments. ^a^ *P* < 0.05 compared to normal control; ^b^ *P* < 0.05 compared to diabetic control; ^c^ *P* < 0.05 compared to glibenclamide; ^d^ *P* < 0.05 compared to HC 100; ^e^ *P* < 0.05 compared to HC 200 (one-way ANOVA followed by Tukey's multiple comparison test).

As there was an increase in plasma insulin levels in *H. cordata*-treated diabetic rats and because of the physiologic importance of insulin-dependent GLUT-2 and GLUT-4 translocation to the cell membrane, attempts have been made to see the effect of *H. cordata* treatment on GLUT-4 level in adipose tissue membrane and GLUT-2 levels in liver and pancreas. In the liver and adipose tissue membrane fractions of diabetic rats, the translocation of GLUT-2 and GLUT-4 was very much reduced when compared with the band density of normal controls. This is quite rational because the deficiency of insulin in the diabetic state would decrease the translocation of GLUT-2 and GLUT-4 from the vesicles to cell membranes. Treatment with *H. cordata* resulted in the significant increase in membrane GLUT-2 and GLUT-4 levels at the dose of 400 mg/kg. However, there was no significant effect on GLUT-2 level in pancreatic cells. The modulation of GLUT-4 and GLUT-2 protein could thus be one of the mechanisms of antidiabetic properties of *H. cordata* (Figures 6 and 7).

### 3.12. Histopathological Studies

**Figure 8:**
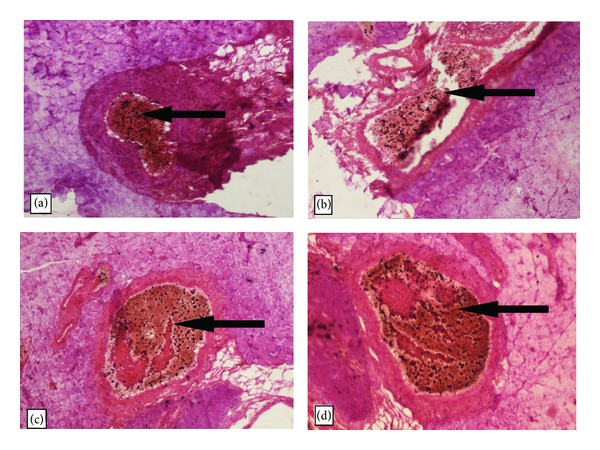
Histopathological view of rat pancreas on treatment with *H. cordata* at 400 mg/kg; p.o.; arrow indicates the location of *β*-cells of pancreas. (a) Normal control. (b) Diabetic control. (c) Diabetic treated with glibenclamide (10 mg/kg; p.o.) and (d) diabetic treated with* H. cordata* (400 mg/kg; p.o.).

The effects of *H. cordata* on pancreatic cells are represented in Figure 8. The pancreas of the normal rats showed normal islets with intact *β*-cells, whereas, in case of diabetic control rats, atrophy of *β*-cells with vascular degeneration in islets was observed. The rats treated with glibenclamide (10 mg/kg; p.o.) and *H. cordata* (400 mg/kg; p.o.) depicted regeneration of *β*-cells which were found to be intact and also preserved islets justifying its protective effect.

## 4. Discussion

Regular administration of ethanolic extract of *H. cordata* for 3 weeks resulted in a significant diminution of FPG level with respect to diabetic rat, which clearly explains its antidiabetic activity. The results demonstrated a dose-dependent effect of *H. cordata* treatment in decreasing FPG. Treatment with *H. cordata* (200 and 400 mg/kg, p.o.) not only lowered the TC, TG, and LDL level, but also enhanced the HDL-cholesterol which is known to play an important role in the transport of cholesterol from peripheral cells to the liver by a pathway termed “reverse cholesterol transport,” and is considered to be a cardioprotective lipid [33]. Decreased levels of BUN, creatinine and elevation in total protein again indicated that *H. cordata* can improve renal and liver function [34].

Dysfunctional mitochondria produce excessive amounts of ROS such as superoxide (O_2_^−^), hydrogen peroxide (H_2_O_2_), and peroxynitrite (ONOO^−^). This, over production of ROS accumulated in the mitochondrial matrix, leads to collapse of mitochondrial membrane potential (ΔΨ_*m*_), decrease in ATP production, and subsequent mitochondrial dysfunction [35]. In line with earlier studies, we also observed that STZ administration produced an increase in the oxidative damage and decreased the antioxidant enzyme activity [36]. Further, there was a significant decrease in mitochondrial function and integrity with the administration of STZ. This effect has been observed in other studies also [37]. *H. cordata* extract attenuated the STZ-induced mitochondrial oxidative stress and stabilized the mitochondrial function and integrity in the pancreatic tissues. It is well accepted that PPAR-*γ* plays a significant role in the pathogenesis of inflammation in several tissues [38]. Moderate amounts of PPAR-*γ* are expressed in pancreatic *β*-cells, which increases in the diabetic state [39], leading to accumulation of intracellular triglyceride. In the present study, the level of expression of PPAR-*γ* was elevated in STZ-induced rats similar to earlier reports. *H. cordata* extract did not cause any change in the STZ-induced inflammation indicating that the extract was probably ineffective against STZ-induced inflammation.

It is reported that STZ injection causes apoptosis in several tissues such as liver, pancreas, and adipose tissues [37]. As a marker of apoptosis, the level of caspase-3 was increased with the STZ injection in all the tissues under the investigation. *H. cordata* extract showed significant lowering of caspase-3 level in pancreatic tissues of STZ-injected rats, indicating its promising effect on STZ-induced apoptosis. It is well documented that caspase-3 is a common product of both extrinsic and intrinsic mediated apoptotic pathways [40]. The effect was also well supported by the histopathological studies showing a considerable regeneration in the *β*-cells of pancreas in rats treated with *H. cordata* 400 mg/kg; p.o. In this context, it is quite impossible to explain the mechanism of *H. cordata* extract in the STZ-induced apoptosis; however further studies may elaborate the plausible antiapoptotic mechanism of *H. cordata* extract in STZ-induced model.

Several tissues are involved in maintaining glucose homeostasis. Among them, liver, pancreatic *β*-cells, and adipose tissue are the most important because they can sense and respond to changing blood glucose levels. Glucose is taken up into the cell through GLUT-2 and GLUT-4 in the plasma membrane of the cells. In pancreatic *β*-cells, glucose is the primary physiological stimulus for insulin secretion [41]. GLUT-2 is known to play more permissive roles, allowing rapid equilibration of glucose across the plasma membrane. However, it is also essential in glucose stimulating insulin signal (GSIS) because normal glucose uptake and subsequent metabolic signaling for GSIS cannot be achieved without GLUT-2. In diabetic subjects, GLUT-2 and GLUT-4 expression is decreased before the loss of GSIS [42]. Our study suggests that the modulation of GLUT-2 and GLUT-4 protein could thus be one of the mechanisms of antidiabetic potential of *H. cordata*.

The study revealed a significant increase in serum insulin level in rats treated with *H. cordata* (especially at 400 mg/kg, p.o.) and glibenclamide as a result of regeneration of pancreatic *β*-cells which were destroyed by streptozotocin [43]. Thus, the antidiabetic effect of *H. cordata* could be attributed to upregulation of GLUT-2 and GLUT-4 protein expressions resulting in potentiation of pancreatic secretion of insulin from existing *β*-cells of islets. Moreover, the study also demonstrated the beneficial role of *H. cordata* in attenuating oxidative stress responsible for mitochondrial dysfunction.

Many works in the literature have shown the antioxidative, anticarcinogenic, antimicrobial, antidiabetic, and anti-inflammatory activities of phenols, flavonoids, and polysaccharides [44]. Among the polyphenols, gallic acid, resveratrol, and quercetin are widely distributed in the plant kingdom and are reported to possess antioxidant and antidiabetic properties [45]. In contrast to our results, *H. cordata* shows anti-inflammatory activity in diabetic condition by improving the level of adiponectins [46]. This discrepancy in the results could be due to the different sets of diabetic condition. The above experiment was performed in the cell lines; however the present study was investigated as an *in vivo* model of diabetes. Moreover, in both studies *H. cordata* showed antiapoptotic effect in pancreatic tissues [46]. Studies on diabetic animal models have shown that quercetin significantly decreases the blood glucose level, plasma cholesterol, and TG in diabetic rats, in dose-dependent manner [47]. Beneficial effects of quercetin in increasing the number of pancreatic islets and protective effect on degeneration of *β*-cells along with facilitation in translocation of GLUT-4 have also been reported in the literature [48, 49]. Thus, the presence of quercetin quantified in *H. cordata* may play a contributing factor to the observed antidiabetic activity via the above mentioned pathways.

## 5. Conclusion

In conclusion, the present study justified the protective role of *H. cordata* on pancreatic *β*-cells under high glucose toxic condition by reducing ROS-induced oxidative stress and apoptosis. These findings demonstrated that *H. cordata* can be employed as a potential pharmaceutical agent against glucotoxicity induced by hyperglycaemia and in oxidative stress associated with diabetes.

## Figures and Tables

**Table 1:** Effect of HC on FPG in streptozotocin-induced diabetic rats.

Group (*n* = 3)	Treatment (dose in mg/kg)	Fasting plasma glucose concentration (mg/dL)			
		1st day	7th day	14th day	21st day
I	NC	85.62 ± 3.8	83.01 ± 3.05	85.40 ± 3.63	80.10 ± 2.92
II	DC	294.72 ± 11.07^a^	319.72 ± 12.41^a^	362.99 ± 13.63^a^	365.15 ± 13.29^a^
III	Glib (10)	327.67 ± 10.7^a^	184.91 ± 6.72^a,b^	152.63 ± 4.67^a,b^	122.62 ± 3.87^b^
IV	HC (100)	271.48 ± 18.87^a^	293.77 ± 16.11^a,c^	320.52 ± 20.89^a,c^	324.64 ± 15.33^a,c^
V	HC (200)	275.07 ± 14.42^a^	322.25 ± 12.68^a,c^	260.82 ± 12.95^a,b,c,d^	190.50 ± 11.36^a,b,c,d^
VI	HC (400)	316.20 ± 15.87^a^	299.68 ± 14.57^a,c^	208.56 ± 11.56^a,b,c,d,^	146.86 ± 8.26^a,b,d,e^

Results are expressed as mean ± SEM, *n* = 6, ^a^ *P* < 0.05 compared to normal control; ^b^ *P* < 0.05 compared to diabetic control; ^c^ *P* < 0.05 compared to glibenclamide; ^d^ *P* < 0.05 compared to HC 100; ^e^ *P* < 0.05 compared to HC 200 (two-way ANOVA followed by Bonferroni posttest).

**Table 2:** Effect of HC on lipid profile of streptozotocin-induced diabetic rats on the 21st day.

Group (*n*=3)	Treatment (dose in mg/kg)	TC (mg/dL)	TG (mg/dL)	VLDL (mg/dL)	HDL-C (mg/dL)	LDL (mg/dL)
I	NC	89.73 ± 3.91	82.67 ± 4.08	16.53 ± 0.81	38.06 ± 1.23	35.13 ± 2.70
II	DC	173.13 ± 6.99^a^	177.41 ± 8.61^a^	35.48 ± 1.72^a^	21.01 ± 0.98^a^	116.63 ± 7.4^a^
III	Glib (10)	96.19 ± 4.26^b^	140.13 ± 8.08^a,b^	28.02 ± 1.61^a,b^	40.96 ± 1.31^b^	27.20 ± 3.60^b^
IV	HC (100)	161.09 ± 7.68^a,c^	161.84 ± 8.70^a^	32.36 ± 1.74^a^	24.13 ± 1.09^a,c^	104.58 ± 8.33^a,c^
V	HC (200)	132.95 ± 7.59^a,b,c,d^	138.15 ± 5.55^a,b^	27.63 ± 1.11^a,b^	31.02 ± 1.6^a,b,c,d^	74.29 ± 6.34^a,b,c,d^
VI	HC (400)	116.09 ± 6.14^b,d^	106.57 ± 4.63^b,c,d,e^	21.31 ± 0.92^b,c,d,e^	35.01 ± 1.76^b,c,d^	59.76 ± 4.51^b,c,d^

Values are expressed as mean ± SEM of 6 animals in each group. One-way ANOVA showed a significant difference in drug treatment between the groups for HC for total cholesterol, triglyceride, very low density lipoprotein (VLDL), HDL-cholesterol (HDL-C), and low density lipoprotein (LDL). ^a^ *P* < 0.05 compared to normal control; ^b^ *P* < 0.05 compared to diabetic control; ^c^ *P* < 0.05 compared to glibenclamide; ^d^ *P* < 0.05 compared to HC 100; ^e^ *P* < 0.05 compared to HC 200 (one-way ANOVA followed by Tukey's multiple comparison test).

**Table 3:** Effect of HC on other biochemical parameters.

Group (*n* = 6)	Treatment (dose in mg/kg)	CRTN (mg/dL) 21 day	BUN (mg/dL) 21 day	TPR (g/dL) 21 day
I	NC	0.641 ± 0.055	21.908 ± 1.392	0.717 ± 0.012
II	DC	1.165 ± 0.135^a^	57.906 ± 4.831^a^	0.615 ± 0.011^a^
III	Glib (10)	0.59 ± 0.019^b^	35.241 ± 2.654^a,b^	0.723 ± 0.012^b^
IV	HC (100)	1.039 ± 0.087^a,c^	52.573 ± 2.474^a,c^	0.614 ± 1.028^a,c^
V	HC (200)	0.792 ± 0.095^b^	44.862 ± 2.143^a,b^	0.646 ± 1.017^a,c^
VI	HC (400)	0.724 ± 0.074^b^	39.745 ± 1.793^a,b,d^	0.697 ± 0.0.02^b,d^

Values are mean ± SEM of 6 animals in each group. One-way ANOVA showed a significant difference in drug treatment between the groups for HC for total creatinine (CRTN), blood urea nitrogen (BUN), and total protein (TPR); ^a^ *P* < 0.05 compared to normal control; ^b^ *P* < 0.05 compared to diabetic control; ^c^ *P* < 0.05 compared to glibenclamide; ^d^ *P* < 0.05 compared to HC 100; ^e^ *P* < 0.05 compared to HC 200 (one-way ANOVA followed by Tukey's multiple comparison test).

**Table 4:** Effect of HC on body weight in streptozotocin-induced diabetic rats.

Group (*n* = 6)	Treatment (dose in mg/kg)	Body weight (g)	
		1st day	21st day
I	NC	147.83 ± 3.42	154.66 ± 4.18
II	DC	149.33 ± 4.15	110.66 ± 5.33^a^
III	Glib (10)	154.83 ± 4.20	145.66 ± 3.89^b^
IV	HC (100)	153.16 ± 5.32	118.5 ± 4.76^a,c^
V	HC (200)	155.33 ± 4.49	124.5 ± 4.02^a,c^
VI	HC (400)	151.83 ± 4.96	136.5 ± 4.48^b^

Values are mean ± SEM of 6 animals in each group. One-way ANOVA reveals that there were significant differences among the experimental groups [*F*(5,30) = 14.12, *P* < 0.05]. ^a^ *P* < 0.05 compared to normal control; ^b^ *P* < 0.05 compared to diabetic control; ^c^ *P* < 0.05 compared to glibenclamide. (One-way ANOVA followed by Tukey's multiple comparison test.)

**Table 5:** Effect of HC on mitochondrial MDA, SOD, CAT, and SDH levels in liver, pancreas, and adipose tissue of diabetic rats.

	Control	Diabetic	GL-10	HC-100	HC-200	HC-400
MDA level (nmol MDA/mg protein)						
Liver	6.31 ± 0.322	10.625 ± 0.411^a^	7.436 ± 0.331^b^	9.95 ± 0.348^a,c^	8.253 ± 0.4^a,b,d^	7.23 ± 0.335^b,d^
Pancreas	3.66 ± 0.29	10.389 ± 0.381^a^	5.071 ± 0.327^b^	8.965 ± 0.396^a,c^	6.082 ± 0.376^a,b,d^	4.71 ± 0.373^b,d^
Adipose tissue	7.092 ± 0.334	13.19 ± 0.356^a^	8.336 ± 0.321^b^	11.93 ± 0.383^a,c^	10.14 ± 0.287^a,b,c,d^	8.218 ± 0.375^b,d,e^

SOD activity (units/min/mg protein)						
Liver	0.152 ± 0.012	0.04 ± 0.007^a^	0.147 ± 0.009^b^	0.076 ± 0.021^a^	0.114 ± 0.015^b^	0.141 ± 0.022^b^
Pancreas	0.384 ± 0.024	0.134 ± 0.019^a^	0.331 ± 0.04^b^	0.17 ± 0.023^a,c^	0.276 ± 0.04^b^	0.319 ± 0.037^b,d^
Adipose tissue	0.512 ± 0.053	0.232 ± 0.018^a^	0.482 ± 0.042^b^	0.272 ± 0.032^a,c^	0.426 ± 0.029^b^	0.46 ± 0.057^b,d^

CAT (units/min/mg protein)						
Liver	21.94 ± 1.415	8.864 ± 1.203^a^	18.733 ± 1.326^b^	11.611 ± 1.523^a,c^	16.631 ± 1.708^b^	20.938 ± 2.674^b,d^
Pancreas	9.605 ± 0.553	3.828 ± 0.505^a^	8.108 ± 1.472^b^	4.505 ± 1.026^a^	5.738 ± 0.644	7.875 ± 1.041^b^
Adipose tissue	13.672 ± 0.896	4.544 ± 0.711^a^	10.91 ± 0.857^b^	7.172 ± 0.784^a,c^	8.325 ± 0.733^a,b^	11.841 ± 0.618^b,d,e^

SDH activity formazan produced (mM/min/mg/protein)						
Liver	1.701 ± 0.066	0.801 ± 0.078^a^	1.581 ± 0.064^b^	0.829 ± 0.35^a,c^	0.883 ± 0.056^a,c^	0.930 ± 0.045^a,c^
Pancreas	2.071 ± 0.058	0.589 ± 0.046^a^	1.882 ± 0.069^b^	0.836 ± 0.033^a,c^	1.591 ± 0.069^a,b,c,d^	1.832 ± 0.067^b,d^
Adipose tissue	2.307 ± 0.187	0.73 ± 0.083^a^	1.814 ± 0.185^b^	0.816 ± 0.056^a,c^	1.151 ± 0.103^a,c^	1.27 ± 0.064^a,c^

All results are expressed as mean ± SEM, *n* = 6, MDA: malondialdehyde, SOD: succinate dehydrogenase, CAT: catalase. ^a^ *P* < 0.05 compared to normal control; ^b^ *P* < 0.05 compared to diabetic control; ^c^ *P* < 0.05 compared to glibenclamide; ^d^ *P* < 0.05 compared to HC 100 and ^e^ *P* < 0.05 compared to HC 200 (one-way ANOVA followed by Tukey's multiple comparison test).
